# Cost–utility analysis of a palliative care program in Colombia

**DOI:** 10.1186/s12904-024-01476-6

**Published:** 2024-07-06

**Authors:** Luisa Rodríguez-Campos, Paul Andres Rodriguez-Lesmes, Analhi Palomino Cancino, Iris del Valle Díaz, Luis Fernando Gamboa, Andrea Castillo Niuman, Juan Sebastián Salas, Gabriela Sarmiento, Jorge Martínez-Bernal, Abel E. González-Vélez

**Affiliations:** 1 Pain and Palliative Medicine. Home Primary Care Unit, Centros Médicos Colsanitas, Calle 163A # 22 08, Bogotá, Colombia; 2 School of Economics, Universidad del Rosario, Bogotá, Colombia; 3 National Medical Coordinator Palliative Care Program, Sanitas Health Insurance, Bogotá, Colombia; 4 Medical Director Primary Home Care Unit, Centros Médicos Colsanitas, Bogotá, Colombia; 5 Epidemiology. Division of Planning, Evaluation, and Knowledge Management, Sanitas CREA, Sanitas Health Insurance, Bogotá, Colombia; 6 Division of Planning, Evaluation, and Knowledge Management, Sanitas CREA, Sanitas Health Insurance, Bogotá, Colombia; 7 Internal Medicine Postgraduate Training, Fundación Universitaria Sanitas, Bogotá, Colombia; 8 Nursing Faculty Dean, Fundación Universitaria Sanitas, Bogotá, Colombia; 9 Preventive Medicine and Public Health medical specialist, Teacher and Researcher Professional of Medicine Department, Universidad de Deusto, Bilbao, Spain

**Keywords:** Palliative Care, Cost–utility analysis, Delivery of Health Care, Insurance providers

## Abstract

**Background:**

The economic assessment of health care models in palliative care promotes their global development. The purpose of the study is to assess the cost-effectiveness of a palliative care program (named Contigo) with that of conventional care from the perspective of a health benefit plan administrator company, Sanitas, in Colombia.

**Methods:**

The incremental cost-utility ratio (ICUR) and the incremental net monetary benefit (INMB) were estimated using micro-costing in a retrospective, analytical cross-sectional study on the care of terminally ill patients enrolled in a palliative care program. A 6-month time horizon prior to death was used. The EQ-5D-3 L questionnaire (EQ-5D-3 L) and the McGill Quality of Life Questionnaire (MQOL) were used to measure the quality of life.

**Results:**

The study included 43 patients managed within the program and 16 patients who received conventional medical management. The program was less expensive than the conventional practice (difference of 1,924.35 US dollars (USD), *P* = 0.18). When compared to the last 15 days, there is a higher perception of quality of life, which yielded 0.25 in the EQ-5D-3 L (*p* < 0.01) and 1.55 in the MQOL (*P* < 0.01). The ICUR was negative and the INMB was positive.

**Conclusion:**

Because the Contigo program reduces costs while improving quality of life, it is considered to be net cost-saving and a model with value in health care. Greater availability of palliative care programs, such as Contigo, in Colombia can help reduce existing gaps in access to universal palliative care health coverage, resulting in more cost-effective care.

**Supplementary Information:**

The online version contains supplementary material available at 10.1186/s12904-024-01476-6.

## Background

Population-based projections indicate that the need for palliative care will increase in the future. Palliative care improves the quality of life of patients and their families dealing with the challenges of a life-threatening illness [1]. So far, the global development of palliative care has been inequitable, and only 14% of the 40 million people in need of palliative care can access it, with most of them living in high-income countries [2, 3]. Cost-effectiveness palliative care models are needed in low- and middle-income countries in order to improve outcomes in terminally ill patients so that an appropriate allocation of resources to finance them can be provided along with an expansion of palliative care provision. The lack of evidence on the cost–utility of palliative care implementation has been a barrier for obtaining administrative support, both in hospitals and among health insurers, and for promoting the expansion of these services [4].

In Colombia, the provision of palliative care services is regulated under Law 1733 of 2014, which states that health benefit plan administrator company (EAPBs, Spanish acronym) must guarantee their members the provision of these services within their network at all levels of care and complexity, in the event of a terminal, chronic, degenerative, or irreversible disease that has an impact on quality of life. Sanitas is one of Colombia’s main EAPBs, insuring 5.8 million people across nation, and has been providing palliative care since February 2017 through a program called Contigo in the cities of Bogotá, Cali, and Medellín. It recently expanded its activities to other cities, such as Barranquilla, Bucaramanga, Ibagué, Pereira, Manizales, Cundinamarca, Armenia, and Cartagena.

This program is based on a patient-centered interdisciplinary care model that can identify and intervene in case of suffering of patients with life-threatening chronic diseases such as cancer, organ failure, neurodegenerative diseases, and severe frailty of older adults when they are in advanced stages with an estimated vital prognosis of 6 months. Patient care is provided through an integrated network that coordinates between the insurance and the specific palliative care providers, delivering outpatient, home, hospital, and hospice services to cover needs based on the patients’ individual complexity while monitoring the quality of life of both patients and caregivers. Between February 2017 and December 2019, the Contigo program cared for 5,236 patients. The purpose of the study is to compare the cost– utility ratio of a palliative care program with conventional care in terms of improving the quality of life of terminally ill adults from the perspective of a health benefit plan administrator company.

## Methods

This is an analytical cross-sectional study that assessed the costs and quality of life associated with the care of patients whose expected survival is approximately 6 months and who are linked to the Sanitas’ Contigo Palliative Care Program in the cities of Bogotá, Medellín, and Cali compared to a group of patients with the same palliative needs but who do not have access to the program owing to its geographical location in Bucaramanga, Fusagasugá, Tunja, and Villavicencio.

The study has two sources of information: the administrative records of the insurer and the characterization surveys. Patients who were eligible to complete the surveys (Supplementary File 1) were identified through active surveillance systems of new patients who meet criteria to enter the Contigo program. Informed consent of the patient and caregiver was subsequently obtained. Then, the date of death of the patients linked to the program was determined (if available) at the time of the analysis, and those patients who died up to 12 months after the date of the survey and had records of using medical or pharmaceutical services during the last 6 months of life were defined as the sample for the analysis. None of the patients included died as a result of COVID-19.

In order to determine the representativeness of the information collected, administrative records were obtained from the insurer on patients who died between November 2019 and April 2021 from causes other than COVID-19 to obtain: (i) a census of patients enrolled in the Contigo program; (ii) a census of patients who could potentially be enrolled in the program because a diagnosis of chronic, degenerative, advanced, or terminal pathologies with a life expectancy of no more than 6 months but were not part of the program. These two censuses could only provide information on costs and not on quality of life.

### Quality of life

To assess the quality of life, two instruments based on the last 15 days prior to the interview were used. The first instrument, the EQ-5D-3 L (version 3) questionnaire, is an instrument used to assess individual perception of health status by evaluating five dimensions (mobility, personal care, daily activities, pain, and anxiety or depression) based on severity levels categorized as follows: 1 = “no problems”; 2 = “some or moderate problems”; and 3 = “many problems.” The derived states are translated into an index ranging from 0 to 1 [5]. The second instrument used, *the* McGill Quality of Life Questionnaire (MQOL), includes 16 aspects related to terminally ill patients’ comprehensive perception of Quality of Life (QoL) in the days prior to their interview, with the best perception of QoL associated with a higher score [6–8].

### Costs

The records from the insurer’s institutional information system were used to calculate costs. The direct costs of patient care were classified as follows: outpatient care, home care, hospitalization, emergencies, and medications [9]. To avoid confusion between the results and variations in the cost of the same technology in different locations and months, the prices of each technology were standardized. To accomplish this, the average price per unit of each medication or benefit in the entire database was estimated, and this value was applied to all patients who registered for this technology.

Two cost pool alternatives were considered: those associated with all the technologies used by the patient and the 40 technologies typically prescribed by palliative care professionals. This second alternative seeks to avoid biases caused by the presence of outliers resulting from high-cost events that fall outside the scope of the program.

The costs are calculated as a monthly average within the last 6 months of life. This value is obtained by dividing the total cost recorded throughout the entire period by six. Subsequently, the values were converted from Colombian pesos (COP) into US dollars (USD) at a rate of $3,119 COP per USD for the year 2019.

### Data analysis

First, the comparability of the Contigo sample with the population affected by the program (Contigo census) was established. For this purpose, a t-test was used to compare the means between samples from the same population, as well as the distribution of costs.

Second, the differences between the Contigo case study and conventional medical management (control) were explored. The outcomes to consider are costs and quality of life indicators. To ensure that the results do not reflect differences caused by potential confounders, the linear regression is estimated:

1 $${Y}_{i} ={\beta }_{0}+{\beta }_{1}{CONTIGO}_{i}+{X}_{i}^{{\prime }}\gamma +{u}_{i}$$

where ${Y}_{i}$ represents the outcomes of interest (EQ-5D, MQoL, Total Costs) based on a binary variable (Contigo) equal to 1 if the patient $i$ belongs to the case study and 0 otherwise. The regression controls for gender, marital status, socioeconomic status, diagnosis, and whether or not they are employed. The ${\beta }_{1}$ coefficient represents the difference in costs between Contigo’s patients and the outcome of interest after controlling for all other factors.

After determining the costs and quality of life associated with palliative care for patients, an assessment of the relationship between quality of life and associated cost was made. To determine whether a program adds value to a society, the incremental cost–utility ratio (ICUR) was estimated. In this case, it represented the cost of improving one quality-adjusted life year (QALY) of the Contigo program (A) versus the conventional medical management of the control group (B):

2 $$ICU{R}_{AB} = \frac{Cos{t}_{A}-Cos{t}_{B}}{QAL{Y}_{A}-QAL{Y}_{B}}$$

Because the QALYs and costs refer to the last 6 months of life, a simple division of the B1 coefficients obtained with Eq. 1 was performed, and the value corresponded to the ICUR per QALY.

In addition, we performed a net benefit regression to obtain estimated of the incremental net monetary benefit (INMB):

3 $$INM{B}_{AB} = \lambda \left(QAL{Y}_{A}-QAL{Y}_{B}\right)-\left(Cos{t}_{A}-Cos{t}_{B}\right)$$

where $\lambda$ corresponds to the willingness-to-pay (WTP) for one additional QALY. The INMB provides a clear interpretation when the ICUR can turn negative, as it is likely with this intervention where it is expected that costs are reduced while health benefits improve [10, 11]. As a measure for $\lambda$ we consider the Colombian cost-effectiveness threshold (CET), which is USD 5,180.8 per QALY [12], and consider alternative values around it for robustness.

Estimates of both ICUR and INMB were obtained with linear regressions [13, 14], and standard errors were computed by bootstrapping the calculation of the differences in outcomes and costs. For data processing Stata, version 16 was used. Detailed regression tables are presented as supplemental material.

## Results

Initially, 87 patients were included in the study, with 48 receiving care through the Contigo program and 39 receiving conventional care. Out of the group of patients receiving care through the program, five patients whose deaths did not occur during the study’s observation period (12 months) were excluded from the final sample. A total of 23 patients were excluded from the conventional care group: 11 because they were included in the Contigo program during the study period and 12 because their deaths did not occur during the study period. The final sample comprised 43 people from the Contigo program and 16 from the control group. Table 1 shows the characteristics of the assessed population.

**Table 1:** Socio-demographic characteristics of the population

	Contigo Program *n* = 43	Control *n* = 16	*P*-val
**Age (mean. sd)**	70.72(14.15)	81.81(12.38)	0.01
**Gender**			
Male	22 (51%)	7 (44%)	0.62
Female	21 (49%)	9 (56%)	0.62
**Marital Status**			
Married	23 (53%)	9 (56%)	0.85
Divorced	15 (35%)	7(44%)	0.54
Single	5 (12%)	0 (0%)	0.16
**Live at**			
Home	40 (93%)	14 (88%)	0.51
Institution/Other	3 (7%)	2 (13%)	0.51
**Education**			
Primary or less/Don’t know	20 (47%)	9 (56%)	0.51
High School	9 (21%)	6 (38%)	0.2
University	14(33%)	1 (6%)	0.04
**Socioeconomics Level**			
Low-Middle	30 (70%)	8 (50%)	0.16
Middle-High	13 (30%)	8 (50%)	0.16
**Labor Participation**			
Worker	16 (37%)	11 (69%)	0.03
House duties	15 (35%)	2 (13%)	0.09
Jubilated	10 (23%)	3 (19%)	0.72
Other	2 (5%)	0 (0%)	0.39
**Primary Diagnosis**			
Cancer	31(72%)	10 (63%)	0.49
COPD	2 (5%)	0 (0%))	0.39
Heart Disease	5 (12%)	4 (25%)	0.21
Severe Frailty	5 (12%)	2 (13%)	0.93
**Recent Hospitalization**			
No	11 (26%)	2 (13%)	0.29
Yes	32 (74%)	14 (88%)	0.29
**Opioid Use**			
Codeine	0 (0%)	1 (6%)	0.1
Hydrocodone	2 (5%)	1 (6%)	0.81
Hydromorphone	8 (19%)	0 (0%)	0.07
Oxycodone	4 (9%)	0 (0%)	0.21
Tapentadol	1 (2%)	0 (0%)	0.55
Methadone	1 (2%)	0 (0%)	0.55
Morphine	14 (33%)	0 (0%)	0.01
Tramadol	3 (7%)	3 (19%)	0.19
None	10 (23%)	11 (69%)	0
**SubQCatheter user**			
No	40 (93%)	13 (81%)	0.19
Yes	3 (7%)	3 (19%)	0.19
**Urinary catheter user**			
No	39 (91%)	10 (63%)	0.01
Yes	4 (9%)	6 (38%)	0.01
**Religion**			
Catholic	37(86%)	15 (94%)	0.42
Agnostic	1 (2%)	0 (0%)	0.55
Other	5 (12%)	1 (6%)	0.55
**Spiritual Support**			
Music	7 (16%)	1 (6%)	0.33
Religious support	13 (30%)	7 (44%)	0.34
Meditation	14 (33%)	4 (25%)	0.58
Supporting Group	3 (7%)	0 (0%)	0.29
Family and friends	6 (14%)	4 (25%)	0.32

**Notes**: The p-value on the last column corresponds to a t-test of difference on sample means

**Fig. 1:**
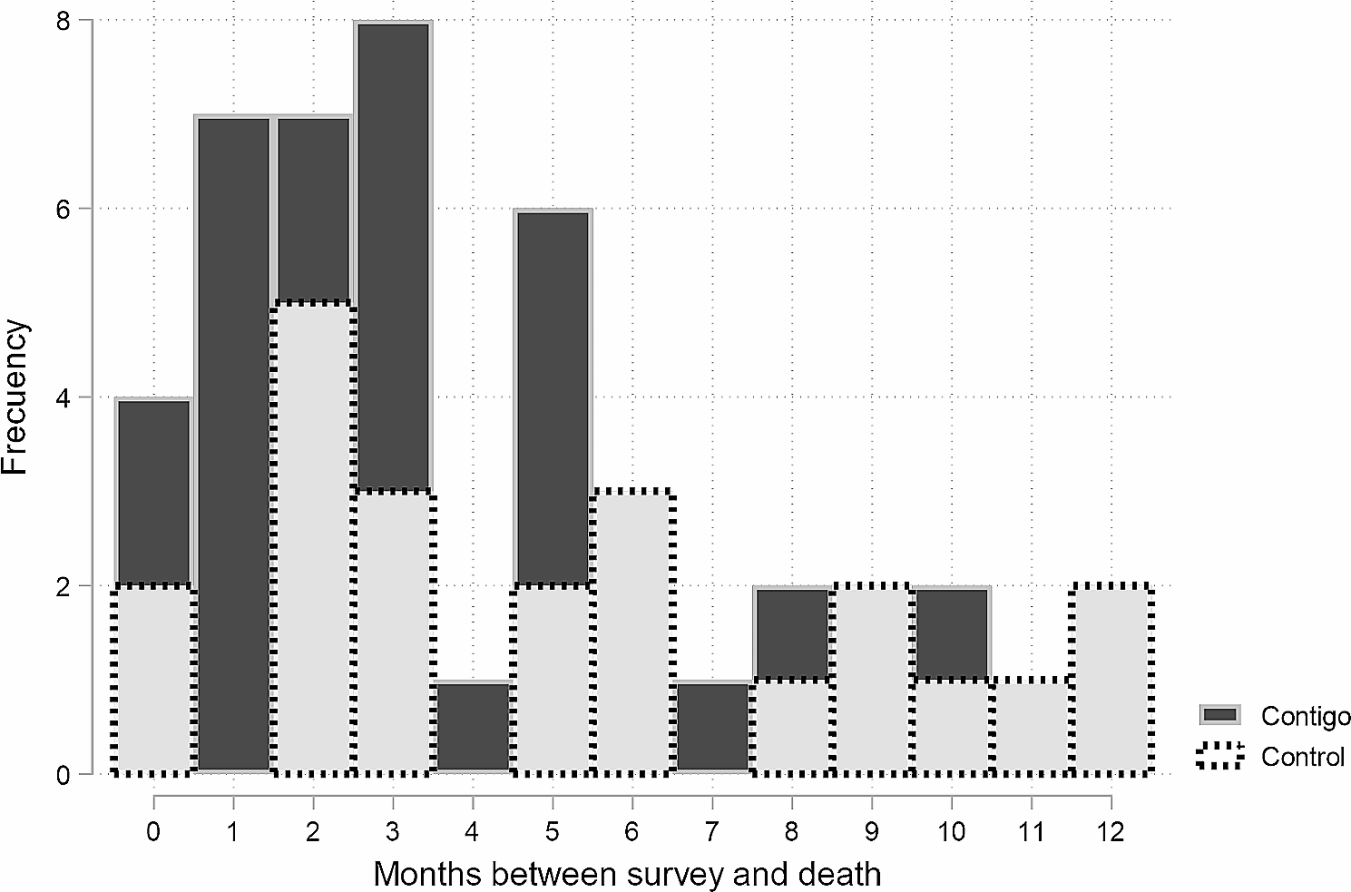
Distribution of Patients according to the date of survey and date of death

Patients are statistically similar in terms of the vast majority of variables considered (*P* < 0.15), but there are some differences in related aspects such as labor participation, use of opioids (such as morphine), and use of a bladder catheter. The socioeconomic conditions of the sample are limited, with the majority of people belonging to the middle and lower strata and a low number of people with higher education levels. Cancer was the most common diagnosis in both groups. Figure 1 shows the time elapsed between the completion of the survey and the death of the patients included in the study. Most of the patients admitted to the Contigo program died within 5 months of completing the survey, while deaths in the control group occurred gradually over the observed time.

In the census of patients who died between November 2019 and April 2021 from causes other than COVID-19, a total of (i) 1,636 individuals enrolled in the Contigo program where found; (ii) 3,551 individuals who could potentially be enroll in the program because a diagnosis of chronic, degenerative, advanced, or terminal pathologies with a life expectancy of no more than 6 months but were not part of the program. The comparison of the study sample and the Contigo patient census (*n* = 1636) (Table 2) reveals similarities in terms of gender and age. The costs related to home care are higher in this study sample when compared to the costs typically observed in the Contigo program. There were no significant differences in cost between patients attended by other providers than home care, neither in the general cost.

**Table 2:** Average standardized monthly costs, 6 months before death – comparison with the full Contigo population

**Panel A. Patient Characteristics**						
	Contigo Sample (*n* = 43)		Contigo Census (*n* = 1636)		Difference of means t-test	
Variable	Mean	sd.	Mean	sd.	Diff	P
Proportion Male	0.47		0.42		0.045	0.553
Age in years	71.67	15.2	73.37	17.12	-1.7	0.538
**Panel B. Total Monthly Cost. 6 months before death**						
	Contigo Sample (*n* = 43)		Contigo Census (*n* = 1636)		Difference of means t-test	
Category	Mean	sd.	Mean	sd.	Diff	P
Outpatient	470.41	1305.95	283.88	1296.49	186.53	0.014
Home Attention	107.03	414.48	96.71	961.01	10.32	0.853
Inpatient	354.6	1691.95	353.65	1730.27	0.95	0.993
Emergencies	26.87	103.15	41.85	179.98	-14.98	0.15
Medicines	330.45	1120.48	446.72	3194.72	-116.27	0.528
Total Cost	1289.36	2593.2	1222.82	4041.71	66.55	0.776

**Fig. 2:**
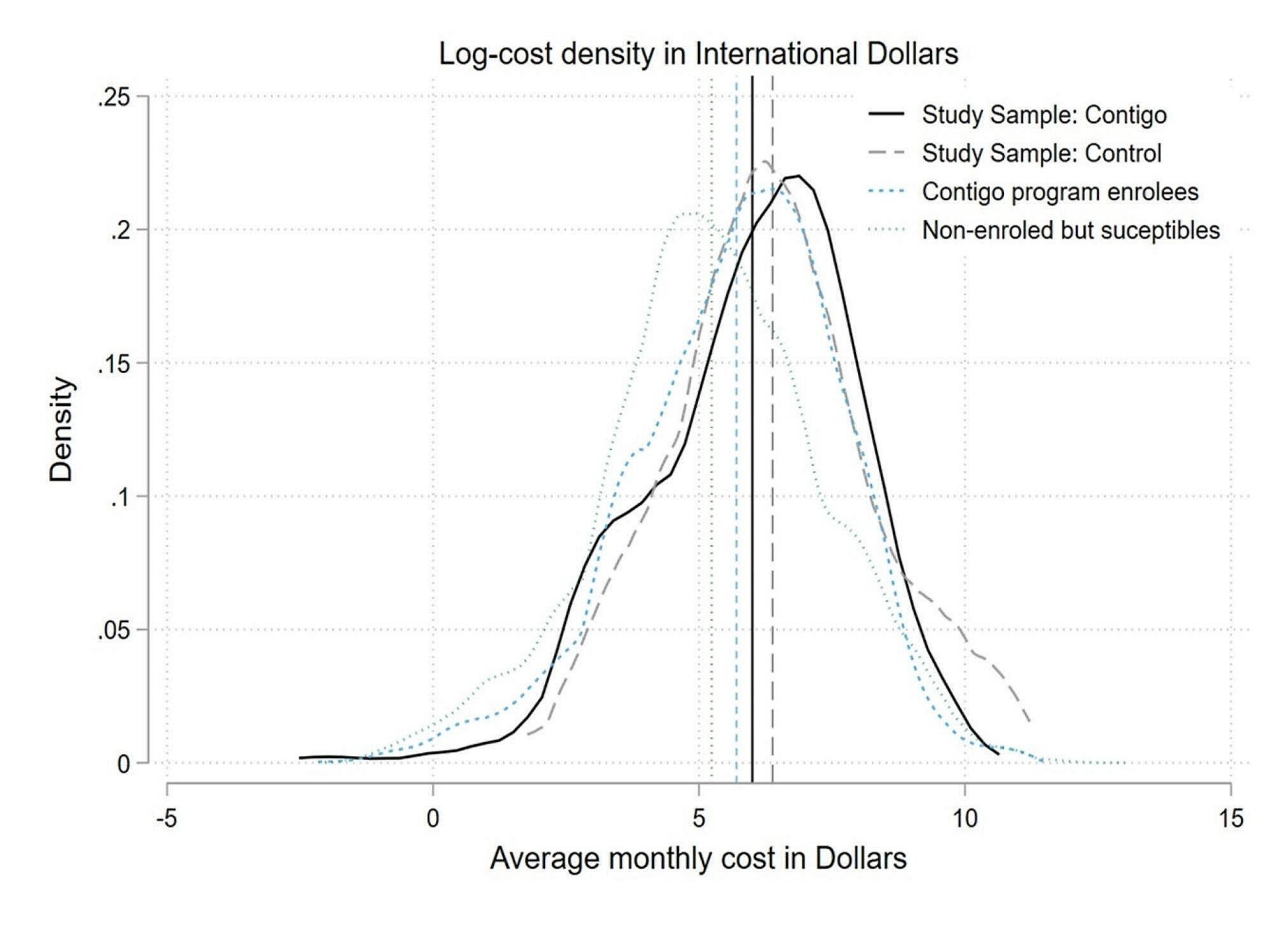
Log-cost density in US dollars

The distribution of the costs under study for the reference populations (sample in the Contigo, sample in conventional care, census Contigo, and census of non-enrolled but susceptible patients) estimated with an Epanechnikov kernel are shown in Fig. 2. Overall, the study found that the Contigo population had a higher average monthly cost than the population that is not part of the program. However, the control sample selected to achieve clinical comparability of cases yielded costs that were much closer to those of the Contigo sample.

When performing the regression of Eq. 1, the estimator of the mean of quality of life perception is higher in patients receiving intervention by the Contigo program than in patients receiving conventional care in both indices (EQ-5D-3 L and MQOL) (Table 3). When considering the individual components of these scales, the EQ-5D-3 L shows variation in the areas of mobility, personal care, and daily activities, while the MQOL shows variation in the psychological and existential areas. In both groups, there is no difference in terms of physical pain.

**Table 3:** Quality of Life 6 months before death (EQ-5D and MQOL in Patients)

	Contigo *n* = 43		Control *n* = 16		Difference of means using an OLS		
	Mean	S.d	Mean	S.d	Diff	SE	*p*-val
**EQ-5D Index**	0.57	0.21	0.31	0.29	0.25	0.07	< 0.01
Mobility	1.86	0.52	2.56	0.63	-0.65	0.16	< 0.01
Personal Care	1.72	0.67	2.5	0.63	-0.72	0.19	< 0.01
Daily Activities	1.91	0.68	2.5	0.63	-0.47	0.2	0.01
Pain	2	0.62	2	0.63	0.04	0.18	0.88
Anxiety or Depression	1.7	0.71	2	0.52	-0.27	0.19	0.19
**MQOL**	**6.58**	**1.63**	**4.8**	**1.54**	**1.55**	**0.47**	**< 0.01**
Physical Symptoms. mean	4.88	2.63	2.04	1.47	2.66	0.7	< 0.01
Physical Welfare. mean	5.86	2.37	4.13	2.78	1.36	0.73	0.04
Psychological Welfare. mean	6.49	2.47	4.78	2.77	1.46	0.75	0.09
Existential Welfare. mean	7.43	1.9	3.47	2.8	3.76	0.64	< 0.01
Support. mean	8.27	1.7	9.59	0.88	-1.5	0.45	< 0.01

**Notes**. Values in the column Diff correspond to the parameter of a linear regression with 57 degrees of freedom between the variables in the rows and a dummy that indicates participation in the program. The regression controls for the presence of a catheter, gender, civil status, socio-economic level, diagnosis, and occupation of the patient

**Fig. 3:**
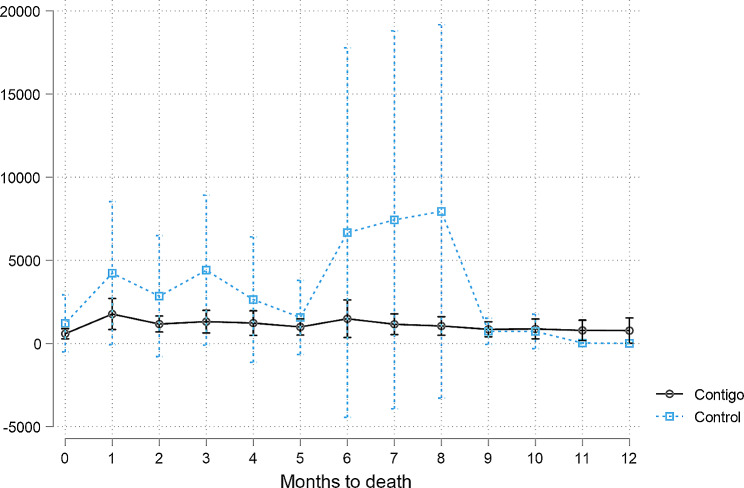
Average monthly costs according to months to death with 95% confidence interval

Figure 3 shows the average monthly cost in the last 12 months of life when comparing the group receiving care through the Contigo program to the group receiving conventional care management. The average costs appear to be higher in the last 8 months before death in the case of conventional management than in the case of Contigo participants. Our assessment focused on the last 6 months of life, which are represented in Table 4. Contigo patients required a value of USD 1,289.36 versus USD 2,783.79 for those patients receiving conventional care. The estimated difference is of − 1,924.4 USD in the Contigo program compared to the costs in conventional management, controlling for characteristics of the patients (*P* = 0.18). The main difference in costs among the Contigo patients is due to the scope of outpatient visits, indicating an opportunity for the health system to save money. In terms of the costs that could be directly associated with the program (Panel B), the difference is USD 145.8 (*P* < 0.01). Additionally, the Contigo program is more expensive in terms of outpatient care and medicines.

**Table 4:** Average standardized monthly costs. 6 months before death

	a. All costs						
	Contigo (*N* = 43)		Control (*N* = 16)		Difference		
Category	Mean	sd.	Mean	sd.	Diff	SE	*p*-val
**Outpatient**	470.41	674.68	91.35	136.65	283.56	110.01	0.01
**Home Attention**	107.03	170.45	2018.65	5580.63	-2004.25	1350.37	0.14
**Inpatient** (Hospital and Hospice)	354.6	624.33	268.03	665.73	-11.72	186.56	0.95
**Emergencies**	26.87	44.36	25.13	55.93	3.58	15.55	0.82
**Medicines**	330.45	962.22	380.63	1284.36	-195.51	317.81	0.54
***Total Cost***	1289.36	1479.63	2783.79	5641.16	-1924.35	1407.81	0.18
	**b. Direct costs: technologies directly linked to Contigo**						
**Category**	**Mean**	**sd.**	**Mean**	**sd.**	**Diff**	**SE**	**p-val**
**Outpatient**	88.5	166.5	2.81	4.63	98.67	33.65	< 0.01
**Home Attention**	46.92	73.82	53.57	80.41	-17.5	24.72	0.48
**Inpatient** (Hospital and Hospice)	6.66	14.6	2.75	8.32	2.54	2.45	0.30
**Emergencies**	1.83	2.17	1.68	4.07	0.47	1.1	0.67
**Medicines**	126.16	166.89	19.43	49.41	61.65	25.93	0.02
***Total Cost***	270.08	256.72	80.24	84.64	145.84	40.77	< 0.01

**Notes**: Own calculations. The values in the Diff column correspond to the parameter of a linear regression with 57 degrees of freedom between the variables in the rows and a dummy indicating participation in the program. The regression controls for the presence of a catheter, gender, civil status, socio-economic level, diagnosis, and occupation of the patient. Values are in USD dollars for 2018. Costs were constructed by adding up standardized costs per calendar month and then averaging those costs for the last six of months of life per patient

The program isnet cost-saving because it improves the quality of life while maintaining the general costs or even reducing them. Considering total costs, the ICUR corresponds to USD − 7,784.14 per QALY [*P* = 0.864]. As the value is negative, it is better to consider the incremental ben benefit. The INMB is of 3204.9 USD [*P* = 0.029, CI 95%: 332.09, 6077.75] using the CET, stable for a wide range of WTP values and significant at the 95% with any WTP above 3.000 USD. If we consider direct costs only, the INMB is still positive and significant.

## Discussion

Expanding palliative care as part of the universal health coverage strategy is one of the priorities undertaken by the Member States of the United Nations in the Sustainable Development Goals by 2030. The public health strategy aimed at promoting the development of palliative care in countries has considered the provision of specialized services to be one of the fundamental pillars, along with access to opioid medications and education. In Colombia by 2019, there were 0.9 services per 100,000 inhabitants which where unequally distributed throughout the national territory, with most services concentrated in large cities [15].

Because the main objective of palliative care is to improve the quality of life of patients and their families, it is important to systematically measure this outcome along the provision of care to assess the impact of the interventions. Cost–utility analyses enable the collection of objective data for advocacy and the expansion of national coverage from the view of the health benefit plan administrator companies, with the aim of favoring the national development of palliative care in the countries. There are several models of palliative care that have been shown to be cost efficient, but studies that analyse the differences in costs between models are needed as an input to assist in decision making for the allocation and expansion of palliative care services [16–19]. Our data address an opportunity for the health system to save money with home palliative care-based models.

The cost impact of the palliative care program is most prominent at the end of life, which corresponds to the highest health care costs in the absence of palliative care. It has been reported in Colombia that the average cost per patient during the last three months and the last month of life respectively represented 52% and 25.6% of the expenditure during the last year of life [20]; an especially for cancer patients where it has been reported to be 60.2% in the last 6 months of life [21]. In this study, the QoL measured by the EQ-5D-3 L and MQOL showed higher levels of average quality of life in patients managed in the Contigo program than in those subjected to conventional medical management, which supports previous findings by González-Vélez et al. [22]. The methodology used does not ensure that the quality of life status can be attributed to program participation and should also be interpreted with caution because the instruments used to measure the quality of life are most likely incapable of adequately measuring some dimensions, such as the psychosocial aspects at the end of life [6, 23]. Micro-costing studies help to raise awareness about the benefits of institutional care programs and can promote the creation or strengthening of teams providing palliative care. The cost of care in the context of the Contigo program is reduced by approximately USD 1,924.35 when patients with a similar life expectancy are considered, even though the direct costs are slightly higher (USD 145,84), implying that costs are compared between those who stayed 6 months or less in the program and those who were under conventional medical management, which served as a control. Other studies are consistent with other findings [16, 19, 24]. In particular, in a systematic review [12], found that palliative care reduces costs by USD 1,285–20,719 for inpatients and by USD 1,000–5,200 for outpatient care. Patients in the Contigo program perceive a higher quality of life, and the conventional care was USD 1,924.35 more expensive on average than the care provided to patients in the Contigo program during the last 6 months of life. Our estimates of the INMB were positive and significant considering the Colombian CET as the WTP measure [12]. Consequently, the Contigo program is considered cost-effective.

This study provides an example of how to measure cost–utility in palliative carebased on the comparison of two cohorts within the same vital timeline, defined retrospectively from death, month by month, ensuring that biasis minimised and there is greater homogeneitybetween the groups being compared, both in terms of clinical condition and perceived quality of life. This can be explained by the fact that they all go through the same stages in the disease’s natural course, resulting in more comparable measurements, even in terms of costs.

A limitation to assess the impact of the program is the high degree of heterogeneity in cost components. In contrast to Gonzalez [22], our study has a clear comparison group based on the date of death and date of survey, and a larger sample. In this sense, has a more homogenous group from which to obtain clear differences. Still, Fig. 3 shows that Contigo has low costs in general, but the high variability of the control costs dilutes the statistical differences between program participants and conventional medical care participants.

The limitations of this study are related to the differentiation of costs between different health suppliers in different regions of the country. However, costs were standardized for this study with a national average, thus allowing for global comparison. The costs obtained from medical bills in a health care system, such as the Colombian one, enable definitive data to be collected in an approximate time span following 90 days of care or even longer, which implies that the timeline for the analysis must account for this time period when assessing the true costs of care for individuals. The identification of patients eligible for palliative care was mainly done using the International Classification of Diseases 10th Revision (ICD10) diagnoses reported in the clinical record, which may indicate the presence of an unidentified population with palliative needs in the health benefit plan administrator company.

Also, we acknowledge the lack of adjustment by cofounders and the small sample size included in this study. We showed that despite the size the study, our results are likely to have internal validity as general characteristics and costs of the sample are similar to the population affiliated to the company. In addition, identification comes from the availability of the services, rather than self-selection. As for external validity, the limitation comes on the capability of other companies to integrate services at the same level of quality and with trained professionals.

## Conclusions

The investment in a palliative care model by a healthcare payer has the potential to provide value to patients while reducing payers’ costs. The attractive estimate of cost-effectiveness makes this model of care a high priority for future researchers to confirm these results and continue to grow the evidence base for the value of palliative care programs throughout the world.

### Electronic supplementary material

Below is the link to the electronic supplementary material.

Supplementary File 1: Questionnaire. Set of questions addressed to the patient and their caregiver on sociodemographic data, state of health and perception of quality of life

Supplementary File 2: Supplementary Tables. Output of regression tables and figures for computing the incremental cost utility ratio and the incremental net monetary benefit

## Data Availability

The datasets used and/or analysed during the current study are available from the corresponding author on reasonable request.

## Abbreviations

EAPB: Health Benefit Plan Administrator Company
MQOL: McGill Quality of Life Questionnaire
QoL: Quality of Life
COP: Colombian pesos
ICUR: Incremental Cost–Utility Ratio
QALY: quality-adjusted life year
USD: United States Dollar
ICD10: Classification of Diseases 10th Revision
