# Supplementary material for: Cost–utility analysis of a palliative care program in Colombia
Source: BMC Palliat Care. 2024 Jul 6;23:165. doi: 10.1186/s12904-024-01476-6 (PMC11227163; doi:10.1186/s12904-024-01476-6)
Supplement: Supplementary file 1 — Questionnaire. Set of questions addressed to the patient and their caregiver on sociodemographic data, state of health and perception of quality of life [file 12904_2024_1476_MOESM1_ESM.docx]

Generated by androdri, Aug 06, 2019 15:16

Questionnaire created by adminpaliativos, Apr 30, 2019 16:03 Last modified by adminpaliativos, Aug 05, 2019 12:10

Shared with:

androdri last edited 7/26/2019 8:44:26 PM

AndreaQuintero (has never been edited)


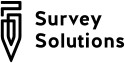
Sections: 4, Sub-subsections: 7, Questions: 146.

Questions with qualifying conditions: 10

Questions with validation conditions 18

Lists: 0

Variables: 0

# Program_evaluation

SURVEY IDENTIFICATION INFORMATION QUESTIONNAIRE DESCRIPTION

PATIENT QUESTIONNAIRE

Sub-subsections: 3, No lists, Questions: 65, Static TEXTs: 4.

PRIMARY CAREGIVER QUESTIONNAIRE

Sub-subsections: 3, No lists, Questions: 59.

CAREGIVER QUESTIONNAIRE 2

Sub-subsections: 1, No lists, Questions: 20.

CLOSING QUESTIONNAIRE

No sub-sections, No lists, Questions: 2.

APPENDIX A - CATEGORIES

LEGEND

*SURVEY IDENTIFICATION INFORMATION*

## QUESTIONNAIRE DESCRIPTION

**Basic information**

*Title* Program_evaluation

SURVEY IDENTIFICATION INFORMATION QUESTIONNAIRE DESCRIPTION

### PATIENT QUESTIONNAIRE

patient_questionnaire

PATIENT QUESTIONNAIRE

#### SOCIO-DEMOGRAPHIC PROFILE

partner_profile_P

| Start time Questionnaire Patient | DATE: CURRENT TIME date_P |
| --- | --- |
| Select user Interviewer | UNIQUE-SELECTION: DX_P COMBO BOX User_questionnaire  01Chief Bogota  02Chief Villavicencio  03Chief Medellin  04Chief Boyaca  05Chief Cali |
| Select user answering the questionnaire | UNIQUE-SELECTION: DX_P COMBO BOX User_respond  01Patient  02Principal_Caregiver  03Caregiver_2 |
| Record the Record Location of the visit. | GPS GPS_P  N  W  A |
| Case/control | UNIQUE-SELECTION: DX_P COMBO BOX Case_control  01Case  02Control |
| Program Entry Date  Control_case == 1 | DATE Ingprog_P |
| Principal Diagnosis | UNIQUE-SELECTION: DX_P COMBO BOX DX_P  01Cancer  02 ALS / Multiple Sclerosis /  Parkinson's / Motor neuron disease  03 Severe Frailty  04 Heart disease  05 COPD  06 HIV  07 Liver Disease |
| ID number | TEXT Num_Doc |

E

| Type of Document Patient | UNIQUE-SELECTION: DX_P COMBO BOX Tip_doc  01CITIZENSHIP ID  02FOREIGNER ID  03UNDERAGE ID  04PASSPORT  05CIVIL REGISTRATION  06TEMPORARY RESIDENCE PERMIT | |
| --- | --- | --- |
| Birthdate | DATE Num_DocP | |
| Gender | SINGLE_SELECT gen_P  01 MALE  02 FEMALE  03 OTHER | |
| Select patient's current marital status | SINGLE_SELECT Civil_status  01SINGLE  02MARRIED  03FREE UNION  04SEPARATE OR  DIVORCED  05WIDOWER | |
| How many people live under the same roof as the patient? | NUMERIC: INTEGER num_pers | |
| select Type of Address  PLACE OF RESIDENCE | SINGLE_SELECT tip_hause  01FAMILY HOME  02GERONTOLOGICAL INSTITUTION  03CHRONIC CARE UNIT  04OTRO | |
| Municipality of residence Patient |  | BOX: COMBO BOX mun_pac  Abejorral (Antioquia)  Acevedo (Huila)  Agrado (Huila)  El Aguila (Valle del Cauca)  Aguachica (Cesar)  Aguada (Santander)  Aguadas (Caldas)  Aguazul (Casanare)  Sativanorte (Boyaca)  Aipe (Huila)  Albania (La Guajira)  Albania (Santander)  Algarrobo (Magdalena)  Algeciras (Huila)  Almaguer (Cauca) Alpujarra (Tolima) |
| Select the type of ethnicity to which the patient belongs. | SINGLE_SELECT ethnicity_P  01AFRO/RAIZAL  02INDIGENOUS  03GYPSIES  04OTRO | |

| Select Patient's Schooling Level | SINGLE_SELECT school_P  01SOME YEARS OF PRIMARY SCHOOL  02HIGH SCHOOL OR SOME YEARS OF HIGH SCHOOL  03ALL HIGH SCHOOL  04TECHNICAL, TECHNOLOGICAL,  SOME YEARS OF  UNIVERSITY  05COMPLETE UNIVERSITY  06DON'T KNOW |
| --- | --- |
| Select Patient's socioeconomic level | UNIQUE-SELECTION: DX_P COMBO BOX stratum_P  01 Level 1  02 Level 2  03 Level 3  04 Level 4  05 Level 5  06 Level 6 |
| What is your approximate monthly income?  Total patient income pesos x month self ==0  It is sure to be well written | NUMERIC: INTEGER ingresop_P |
| What is the total household income?  Total Household income in pesos x month ingresop_P>0  It is sure to be well written | NUMERIC: INTEGER ingreso_P |
| Did you perform any of the following activities during the last two weeks?  Check all options that apply | MULTIPLE-SELECTION acti_P  01WORKING  02LOOKING FOR A JOB  03STUDYING  04HOUSEWORK  05RETIRED WITHOUT PENSION  06PENSIONER  07 UNABLE TO WORK  08OTHER? |
| What other activity do I do  Fill in Field, if in the previous question you selected OTHER WHICH? | TEXT acti_P_other |
| Approximately how many hours per week do you work?  Fill in Field, if in the previous question you selected WORKING - Hours week | NUMERIC: INTEGER time_job_P |
| Have you spent at least one night in a hospital in the last 6 months? | SINGLE_SELECT time_hospital  01YES  02NO |

| Do you take any of the following medications?  Select all the options that apply. | MULTIPLE-SELECTION medica  01CODEINE  02TRAMADOL  03TAPENTADOL  04MORPHINE  05HIDROMORPHONE  06HYDROCODONE  07OXICODONE  08BUPRENORPHINE  09METADONE  10FENTANYL  11NONE |
| --- | --- |
| Does the patient have a subcutaneous catheter? | SINGLE_SELECT catheter_SUB  01YES  02NO |
| Does the patient have a bladder catheter? | SINGLE_SELECT Probe_Versi  01 YES  02NO |
| Does the patient have a gastrostomy? | SINGLE_SELECT gastro  01 YES  02NO |
| Does the patient have a tracheostomy? | SINGLE_SELECT Traque_SUB  01 YES  02NO |
| Does the patient have injuries resulting from dependency? | SINGLE_SELECT Depen_P  01 YES  02NO |
| Religious belief Patient | SINGLE_SELECT reli_P  01CATOLIC  02EVANGELIC  03JEHOVAH'S WITNESS  04NOT A BELIEVER 05OTHER WHICH ONE? |
| Religious belief Patient_ Other  Diligence of the patient's other religious beliefs  reli_P == 5 | TEXT reli_P_other |
| In times of crisis or distress, select the activities you do to improve your emotional state?  Spiritual resource (Nominal, Multinomial) Practices, objects or relationships to which people turn for help in times of crisis or distress. | MULTIPLE-SELECTION Rec_Espi_P  01MUSIC  02ORATION  03MEDITATION  04SUPPORT GROUPS  05SACRED SCRIPTURE (E.G.  BIBLE, TORAH)  06POESY  07SACRAMENTAL PRACTICES  (E.G. COMMUNION, MASS)  08FAMILY  09FRIENDS  10OTHER WHICH? |
| What other spiritual activity does the patient practice? Fill in field, if selected in the previous question OTHER WHICH? | TEXT recur_espi |

I

I

E

I

I PATIENT QUESTIONNAIRE

#### HEALTH STATUS EQ5D

status_health

| Start time EQ5D | DATE: CURRENT TIME dateEQ5D_P | |
| --- | --- | --- |
| We would like you to tell us, in your opinion, how good or bad is your state of health today; On a scale of 0 to 100, where 0 is the worst state of health you can imagine and 100 is the best state of health, what would be your rating? self>=0 && self<=100  The number must be between 0 and 100 | NUMERIC: INTEGER EQ5D_health_P | |
| Mobility | SINGLE_SELECT EQ5D_mobility_P  01I have no problem walking  02I have some problems walking  03I have to be in bed | |
| Personal care | SINGLE_SELECT EQ5D_care_P  01I have no problem with personal care  02I have some problems in bathe or dressing myself.  03I am unable to bath or dress myself. | |
| Daily activities (e.g., work, study, housework, family activities or leisure activities). | SINGLE_SELECT  01I have no problem performing  my daily activities  02I have some problems performing my daily activities.  03I am unable to perform my daily activities. | EQ5D_activities_P |
| Pain/Discomfort | SINGLE_SELECT  01I have no pain or discomfort  02I have moderate pain or discomfort  03I have a lot of pain or discomfort | EQ5D_pain_P |
| Anxiety/Depression | SINGLE_SELECT  01I am not anxious or depressed  02I am moderately anxious or depressed  03I am very anxious or depressed | EQ5D_anxiety_P |

V1

M1

PATIENT QUESTIONNAIRE

#### QUALITY OF LIFE MQOL

MQOL_Patient

DATE: CURRENT TIME

dateMQOL_P

Start time MQOL

STATIC TEXT

*Questions begin with a statement followed by two opposite answers. The numbers extend from one end to the opposite. Please circle a number from 0 to 10 that best indicates your answer. There are no right or wrong answers. Honest answers will be most helpful.*

STATIC TEXT

*PART A: In this series of questions, we begin by asking you to answer the following question about how you have been feeling over the past two days.*

SELECTION-ONLY

quality_P

00

01

02

03

04

05

06

07

08

09

10

STATIC TEXT

Considering all parts of your life - physical,

emotional, social, spiritual, and economic - MY

QUALITY OF LIFE IN THE LAST TWO DAYS ha

been:

Ask him/her for a number from 0 to 10 where 0 is very bad and 10 is excellent.

elente

I

quality_P>=0 && quality_P<11

V1

The number must be between 0 and 10

M1

0

Very bad

1

2

3

4

5

6

7

8

9

10

Excellent

*You must continue the series with PART B. Please name three symptoms or physical problems that have been the most severe for you during the past two days. (Examples include: pain, fatigue, weakness, nausea, vomiting, constipation, diarrhea, trouble sleeping, shortness of breath, loss of appetite, profuse sweating, and immobility. Feel free to refer to others if necessary). Select the number that best indicates how severe each problem has been during the past two days. If, over the two days, you have had no symptoms or problems, or if you have had only one or two, answer only for the ones you have named and we will continue with Part C.*

| 1. For the past two (2) days, a troublesome symptom has been:  Select one of the problematic symptoms of greater intensity. | UNIQUE-SELECTION: DX_P COMBO BOX sinto1_P  01PAIN  02FATIGUE  03DEBILITY  04NAUSEA  05VOMIT  06CONSTIPATION  07DIARREA  08PROBLEMS SLEEPING  09SHORT BREATH  10LOSS OF APPETITE  11CONFUSED SWEAT  12INMOBILITY  13OTHER WHICH_1 |
| --- | --- |
| What is the symptom? syntho1_P==13 | TEXT sinto1Nom_P |
| How problematic has this symptom been?  Ask him for a number from 0 to 10 where 0 is no problem and 10 is a tremendous problem. | SINGLE_SELECT symptomFeel1_P  000 No problem  011  022  033  044  055  066  077  088  099  1010 Tremendous Problem |

I

E

I

| 2. For the past two (2) days, a troublesome symptom has been:  Select one of the problematic symptoms of greater intensity. | UNIQUE-SELECTION: DX_P COMBO BOX sinto2_P  01PAIN  02FATIGUE  03DEBILITY  04NAUSEA  05VOMIT  06CONSTIPATION  07DIARREA  08PROBLEMS SLEEPING  09SHORT BREATH  10LOSS OF APPETITE  11CONFUSED SWEAT  12INMOBILITY  13OTHER WHICH_2 |
| --- | --- |
| What is the symptom? sinto2_P==13 | TEXT sinto2Nom_P |
| How problematic has this symptom been?  Ask him for a number from 0 to 10 where 0 is no problem and 10 is a tremendous problem. | SINGLE_SELECT symptomFeel2_P  000 No problem  011  022  033  044  055  066  077  088  099  1010 Tremendous Problem |
| 3. For the past two (2) days, a troublesome symptom has been:  Select one of the problematic symptoms of greater intensity. | UNIQUE-SELECTION: DX_P COMBO BOX sinto3_P  01PAIN  02FATIGUE  03DEBILITY  04NAUSEA  05VOMIT  06CONSTIPATION  07DIARREA  08PROBLEMS SLEEPING  09SHORT BREATH  10LOSS OF APPETITE  11CONFUSED SWEAT  12INMOBILITY  13OTHER WHICH_3 |
| What is the symptom? sinto3_P==13 | TEXT sinto3Nom_P |

E

| How problematic has this symptom been?  Ask him for a number from 0 to 10 where 0 is no problem and 10 is a tremendous problem. | SINGLE_SELECT  000 No problem  011  022  033  044  055  066  077  088  099  1010 Tremendous Problem | symptomFeel3_P |
| --- | --- | --- |
| For the last two (2) days I have been feeling Physically:  Ask him/her for a number from 0 to 10 where 0 is physically terrible and  10 is physically well sintomas_g>=0 && sintomas_g<11  The number must be between 0 and 10 | SINGLE_SELECT  000 Physically Terrible  011  022  033  044  055  066  077  088  099  1110 Physically Well | sintomas_g |

*Now we will begin PART C. Please select the number that best describes your feelings and thoughts during the last two days.*

| For the past two days, I have been depressed:  Ask him/her for a number from 0 to 10 where 0 is "not at all" and 10 is "extremely" depresion>=0 && depresion<11  The number must be between 0 and 10 | SINGLE_SELECT  000 None  011  022  033  044  055  066  077  088  099  1010 Extremely | depression |
| --- | --- | --- |
| For the past two days, I have been nervous or worried:  Ask him/her for a number from 0 to 10 where 0 is "not at all" and 10 is "extremely" depresion>=0 && depresion<11  The number must be between 0 and 10 | SINGLE_SELECT  000 None  011  022  033  044  055  066  077  088  099  1010 Extremely | concern |

| During the last two days, how long have you felt sad?  Ask him/her for a number from 0 to 10 where 0 is "not at all" and 10 is "extremely" depresion>=0 && depresion<11  The number must be between 0 and 10 | SINGLE_SELECT  000 Never  011  022  033  044  055  066  077  088  099  1010 Always | sadness |
| --- | --- | --- |
| During the last two days, when I thought about the future:  Ask him/her for a number from 0 to 10 where 0 is "not at all" and 10 is "extremely" depresion>=0 && depresion<11  The number must be between 0 and 10 | SINGLE_SELECT  000 I was not afraid  011  022  033  044  055  066  077  088  099  1010 I was terrified | future |
| For the past two days, my life has been:  Ask him/her for a number from 0 to 10 where 0 is "not at all" and 10 is "extremely" depresion>=0 && depresion<11  The number must be between 0 and 10 | SINGLE_SELECT  000 No Meaning and No Purpose  011  022  033  044  055  066  077  088  099  1010 With a lot of sense and Purpose | life |
| For the past couple of days, when I have thought about my whole life, I have felt that in trying to achieve my goals:  Ask him/her for a number from 0 to 10 where 0 is "not at all" and 10 is "extremely" depresion>=0 && depresion<11  The number must be between 0 and 10 | SINGLE_SELECT  000 I have not made any progress  011  022  033  044  055  066  077  088  099  1010 I have made full progress | goals |

| During the last two days, when I thought about my life, I have felt that until today my life has been:  Ask him/her for a number from 0 to 10 where 0 is "not at all" and 10 is "extremely" depresion>=0 && depresion<11  The number must be between 0 and 10 | SINGLE_SELECT  000 Completely in vain  011  022  033  044  055  066  077  088  099  1010 Very valuable, it was worth it. | life_value |
| --- | --- | --- |
| For the past two days, I have felt that:  Ask him/her for a number from 0 to 10 where 0 is "not at all" and 10 is "extremely" depresion>=0 && depresion<11  The number must be between 0 and 10 | SINGLE_SELECT  000 I have no control over my life  011  022  033  044  055  066  077  088  099  1010 I have complete control over my life | life_control |
| As a person, I have felt very good the last two days.  Ask him/her for a number from 0 to 10 where 0 is "not at all" and 10 is "extremely" depresion>=0 && depresion<11  The number must be between 0 and 10 | SINGLE_SELECT  000 I do not agree at all  011  022  033  044  055  066  077  088  099  1010 I completely agree | sensation_per |
| For me, the last two days have been:  Ask him/her for a number from 0 to 10 where 0 is "not at all" and 10 is "extremely" depresion>=0 && depresion<11  The number must be between 0 and 10 | SINGLE_SELECT  000 One load / one weight  011  022  033  044  055  066  077  088  099  1010 A Blessing/Gift | percep_2days |

M1 I

V1

M1

### PRIMARY CAREGIVER QUESTIONNAIRE

CP_Questionnaire

SINGLE-SELECTION

world_2days

10

00

01

02

03

04

05

06

07

08

09

SINGLE-SELECTION

support

00

01

02

03

04

05

06

07

08

09

10

For the past two days, the world has been:

For the past two days, I have been feeling

supported:

Ask him/her for a number from 0 to 10 where 0 is "nothing" and 10 is "extreme".

amente"

I

depresion>=0 && depresion<11

V1

The number must be between 0 and 10

M1

0

An Impersonal and Insensitive Place

1

2

3

4

5

6

7

8

9

10

Attentive and responsive to my

needs

Ask him/her for a number from 0 to 10 where 0 is "nothing" and 10 is "extreme".

amente"

I

depresion>=0 && depresion<11

V1

The number must be between 0 and 10

M1

Nothing

0

1

2

3

4

5

6

7

8

9

10

Completely

PRIMARY CAREGIVER QUESTIONNAIRE

#### SOCIO-DEMOGRAPHIC PROFILE

partner_profile_CP

| Start time main caregiver | DATE: CURRENT TIME time_CP |
| --- | --- |
| Document Number Primary_Caregiver | TEXT Num_Doc_CP |
| Primary Caregiver Document Type | UNIQUE-SELECTION: DX_P COMBO BOX Tip_doc_CP  01CITIZENSHIP ID  02FOREIGNER ID  03UNDERAGE ID  04PASSPORT  05CIVIL REGISTRATION  06TEMPORARY RESIDENCE PERMIT |
| Primary Caregiver's Date of Birth | DATE F_nac_CP |
| Select Gender of Primary Caregiver | SINGLE_SELECT gen_CP  01MALE  02 FEMALE  03OTHER |

| Select current marital status of primary caregiver | SINGLE_SELECT CP_civil_status  01SINGLE  02MARRIED  03FREE UNION  04SEPARATE OR  DIVORCED  05WIDOWER | |
| --- | --- | --- |
| Enter Primary Caregiver's Phone Number | TEXT corr_ele_CP | |
| Does the primary caregiver live with the patient? | SINGLE_SELECT Vive_CP  01YES  02NO | |
| How long is the travel time from your home to the patient's home?  Data in Minutes Vive_CP == 2 | NUMERIC: INTEGER Time_desp_CP | |
| Municipality of residence Primary Caregiver |  | BOX: COMBO BOX mun_CP  Abejorral (Antioquia)  Acevedo (Huila)  Agrado (Huila)  El Aguila (Valle del Cauca)  Aguachica (Cesar)  Aguada (Santander)  Aguadas (Caldas)  Aguazul (Casanare)  Sativanorte (Boyaca)  Aipe (Huila)  Albania (La Guajira)  Albania (Santander)  Algarrobo (Magdalena)  Algeciras (Huila)  Almaguer (Cauca) Alpujarra (Tolima) |
| Select Schooling Level of Primary Caregiver | SINGLE_SELECT school_CP  01SOME YEARS OF PRIMARY SCHOOL  02HIGH SCHOOL OR SOME YEARS OF HIGH SCHOOL  03ALL HIGH SCHOOL  04TECHNICAL, TECHNOLOGICAL,  SOME YEARS OF  UNIVERSITY  05COMPLETE UNIVERSITY  06DON'T KNOW | |
| Select Socioeconomic Status of Primary Caregiver | SINGLE_SELECT stratum_CP  01 Level 1  02 Level 2  03 Level 3  04 Level 4  05 Level 5  06 Level 6 | |

I

| What is your approximate monthly income?  Total income of primary caregiver pesos x month | NUMERIC: INTEGER ingresop_CP |
| --- | --- |
| In addition to caregiving, do you do any of the following activities during the last two weeks?  Check all options that apply | MULTIPLE-SELECTION acti_CP  01WORKING  02LOOKING FOR A JOB  03STUDYING  04HOUSEWORK  05RETIRED WITHOUT PENSION  06PENSIONER  07IUNABLE TO WORK  08OTHER? |
| What other activity do I do?  Fill in Field, if in the previous question you selected OTHER WHICH? | TEXT acti_other_CP |
| Approximately how many hours per week do you work?  Fill in Field, if in the previous question you selected WORKING - Hours per week | NUMERIC: INTEGER time_job_CP |
| How long have you been caring for the Patient?  Enter data in days | NUMERIC: INTEGER Days_CP |
| How many hours per week do you spend caring for/companying the patient?  Fill in data Hours- Week | NUMERIC: INTEGER time_cuid_CP |
| Does the primary caregiver receive any type of remuneration for the care? | SINGLE_SELECT payment_CP  01YES  02NO |
| What type of payment do you receive?  Select corresponding option - species = (e.g. market, housing, food) payment_CP == 1 | SINGLE_SELECT paymentsi_CP  01YES, In cash  0 YES, In-Kind  03 Both |
| Indicate in pesos what you receive, if in-kind, how much would it cost?  How much would it cost you to acquire it? | NUMERIC: INTEGER payment_en_CP |
| Religious belief Primary Caregiver | SINGLE_SELECT reli_CP  01CATOLIC  02EVANGELIC  03JEHOVAH'S WITNESS  04NOT A BELIEVER 05OTHER WHICH ONE? |
| Religious belief Primary Caregiver_ Other  Diligence of the patient's other religious beliefs  reli_CP == 5 | TEXT reli_other_CP |

| Spiritual resource (Nominal, Multinomial) Practices, objects or relationships to which people turn for help in times of crisis or distress_ Primary caregiver | MULTIPLE-SELECTION Rec_Espi_CP  01MUSIC  02ORATION  03MEDITATION  04SUPPORT GROUPS  05SACRED SCRIPTURE (E.G.  BIBLE, TORAH)  06POETRY  07SACRAMENTAL PRACTICES  (E.G. COMMUNION, MASS)  08FAMILY  09FRIENDS  10OTHER WHICH? |
| --- | --- |
| What other spiritual activity does the Primary Caregiver practice?  Fill in field, if selected in the previous question OTHER WHICH? | TEXT recur_espi_CP |
| Relationship of the primary caregiver to the patient | SINGLE_SELECT CP_relationship  01 SPOUSE  02SONS/DAUGHTERS/STEPCHILDREN  03NIETO(A)  04FATHER/MOTHER/FATHER-IN-LAW  05SIBLING/STEPBROTHER/STEPBROTHER SON-IN-LAW/DAUGHTER-IN-LAW  06ANOTHER FAMILY MEMBER  07SERVICE EMPLOYEE  08HIRED TO CARE FOR  09OTHER NON-FAMILY |
| Has the primary caregiver received any training in the role? | SINGLE_SELECT between_CP  01YES  02NO |
| What is the primary caregiver's level of training according to the following options?  Primary Caregiver Information between_CP ==1 | SINGLE_SELECT level_between_CP  01BASIC (POSITION CHANGES AND CARE  OF THE SKIN)  02INTERMEDIATE (PHYSICAL THERAPY,  RESPIRATORY,  MEDICATIONS AND  SIMPLE CURES OF  WOUNDS)  03 ADVANCED (DEVICE HANDLING)  VESICAL CATHETHER OR GASTROSTOMY,  ENTERAL NUTRITION AND  COMPLEX WOUND MANAGEMENT |
| Are you a person with a nursing degree or similar?  (e.g. nursing assistant)  Primary Caregiver Information | SINGLE_SELECT PC_Title  01YES  02NO |

PRIMARY CAREGIVER QUESTIONNAIRE

Greeting_Status_CP

DATE: CURRENT TIME

dateEQ5D_CP

Start time EQ5D

#### HEALTH STATUS EQ5D

| We would like you to tell us, in your opinion, how good or bad is your state of health today; on a scale of 0 to 100, where 0 is the worst state of health you can imagine and 100 is the best state of health, what would be your rating?  EQ5D_CP_health>=0 && EQ5D_CP_health<=100  The number must be between 0 and 100 | NUMERIC: INTEGER EQ5D_health_CP |
| --- | --- |
| Mobility | SINGLE_SELECT EQ5D_mobility_CP  01I have no problem walking  02I have some problems walking  03I have to be in bed |
| Personal care | SINGLE_SELECT EQ5D_care_CP  01I have no problem with personal care  02I have some problems in bathe or dressing myself.  03I am unable to bathe or dress myself. |
| Daily activities (e.g., work, study, housework, family activities or leisure activities). | SINGLE_SELECT EQ5D_activities_CP  01I have no problem performing  my daily activities  02I have some problems performing my daily activities.  03I am unable to perform my daily activities. |
| Pain/Discomfort | SINGLE_SELECT EQ5D_pain_CP  01I have no pain or discomfort  02I have moderate pain or discomfort  03I have a lot of pain or discomfort |
| Anxiety/Depression | SINGLE_SELECT EQ5D_anxiety_CP  01I am not anxious or depressed  02I am moderately anxious or depressed  03I am very anxious or depressed |

V1

M1

PRIMARY CAREGIVER QUESTIONNAIRE

#### ZARIT TEST

OVERLOAD_SCALE_CP

E relation_CP!=8

| Start time Zarit | DATE: CURRENT TIME zarit_CP |
| --- | --- |
| Do you feel that your family member is asking for more help than he/she really needs? | SINGLE_SELECT CP_Help  00Never  01Rarely  02Sometimes  03Several times  04Almost Always |

| Do you feel that because of the time you spend with your family member you no longer have enough time for yourself? | SINGLE_SELECT  00Never  01Rarely  02Sometimes  03Several times  04Almost Always | Help2_CP |
| --- | --- | --- |
| Do you feel tense when you have to care for your family member and attend to other responsibilities as well? | SINGLE_SELECT  00Never  01Rarely  02Sometimes  03Several times  04Almost Always | Help3_CP |
| Are you ashamed of your family member's behavior? | SINGLE_SELECT  00Never  01Rarely  02Sometimes  03Several times  04Almost Always | Help4_CP |
| Do you feel angry when you are near your family member? | SINGLE_SELECT  00Never  01Rarely  02Sometimes  03Several times  04Almost Always | Help5_CP |
| Do you think that the current situation negatively affects your relationship with friends and other members of your family? | SINGLE_SELECT  00Never  01Rarely  02Sometimes  03Several times  04Almost Always | Help6_CP |
| Do you fear for the future of your family member? | SINGLE_SELECT  00Never  01Rarely  02Sometimes  03Several times  04Almost Always | Help7_CP |
| Do you feel that your family member depends on you? | SINGLE_SELECT  00Never  01Rarely  02Sometimes  03Several times  04Almost Always | Help8_CP |
| Do you feel overwhelmed when you have to be with your family member? | SINGLE_SELECT  00Never  01Rarely  02Sometimes  03Several times  04Almost Always | Help9_CP |

| Do you feel that your health has suffered as a result of caring for your family member? | SINGLE_SELECT  00Never  01Rarely  02Sometimes  03Several times  04Almost Always | Help10_CP |
| --- | --- | --- |
| Do you feel that you do not have the private life you would like because of your family member? | SINGLE_SELECT  00Never  01Rarely  02Sometimes  03Several times  04Almost Always | Help11_CP |
| Do you think your social life has been affected by having to care for your family member? | SINGLE_SELECT  00Never  01Rarely  02Sometimes  03Several times  04Almost Always | Help12_CP |
| Do you feel uncomfortable inviting friends over because of your family member? | SINGLE_SELECT  00Never  01Rarely  02Sometimes  03Several times  04Almost Always | Help13_CP |
| Do you think your family member expects you to take care of him or her, as if you were the only person he or she can count on? | SINGLE_SELECT  00Never  01Rarely  02Sometimes  03Several times  04Almost Always | Help14_CP |
| Do you feel that you do not have enough money to care for your family member in addition to your other expenses? | SINGLE_SELECT  00Never  01Rarely  02Sometimes  03Several times  04Almost Always | Help15_CP |
| Do you feel that you will be unable to care for your family member for much longer? | SINGLE_SELECT  00Never  01Rarely  02Sometimes  03Several times  04Almost Always | Help16_CP |
| Do you feel that you have lost control over your life since your family member's illness manifested itself? | SINGLE_SELECT  00Never  01Rarely  02Sometimes  03Several times  04Almost Always | Help17_CP |

SINGLE-SELECTION

Help18_CP

00

01

02

03

04

SELECTION-ONLY

Help19_CP

00

01

02

03

04

SINGLE-SELECTION

Help20_CP

00

01

02

03

04

SELECTION-ONLY

Help21_CP

00

01

02

03

04

SELECTION-ONLY

Help22_CP

00

01

02

03

04

Would you like to be able to take care of your family member?

to other people?

Do you feel unsure about what to do?

with your family member?

Do you feel that you should be doing more than you do for

your family member?

Do you think you could take care of your family member better than you could?

who does it?

In general: Do you feel very overburdened by having

than taking care of your family member?

Never

Rarely

Sometimes

Quite often

Almost always

Never

Rarely

Sometimes

Quite often

Almost always

Never

Rarely

Sometimes

Quite often

Almost always

Never

Rarely

Sometimes

Quite often

Almost always

Never

Rarely

Sometimes

Quite often

Almost always

|  |  |
| --- | --- |

ICLOSING QUESTIONNAIRE

CLOSING QUESTIONNAIRE

CLOSING QUESTIONNAIRE
