# Supplementary material for: Cost–utility analysis of a palliative care program in Colombia
Source: BMC Palliat Care. 2024 Jul 6;23:165. doi: 10.1186/s12904-024-01476-6 (PMC11227163; doi:10.1186/s12904-024-01476-6)
Supplement: Supplementary file 2 — Supplementary Tables. Output of regression tables and figures for computing the incremental cost utility ratio and the incremental net monetary benefit [file 12904_2024_1476_MOESM2_ESM.docx]

**Appendix Table 1. Output of regression estimates**

|  | (1) | (2) | (3) | (4) | (5) | (6) |
| --- | --- | --- | --- | --- | --- | --- |
| Independent variables | **EQ-5D** | **MQoL** | **Total Cost - ALL** | **Total Cost - Direct** | **Net Benefit - All costs** | **Net benefit - Direct costs** |
|  |  |  |  |  |  |  |
| Contigo | 0.247*** | 1.548*** | -1924.4 | 145.8*** | 3204.9** | 1134.7** |
|  | (0.0825) | (0.456) | (1407.8) | (40.77) | (1255.5) | (440.3) |
|  |  |  |  |  |  |  |
| SubQCatheter user | -0.193 | -1.131** | -1928.7* | -147.2** | 929.5 | -852.1 |
|  | (0.127) | (0.478) | (1079.5) | (56.08) | (1185.1) | (641.2) |
|  |  |  |  |  |  |  |
| Male | -0.0539 | -0.785* | -51.27 | -12.08 | -227.9 | -267.1 |
|  | (0.0826) | (0.449) | (981.2) | (78.40) | (846.2) | (447.7) |
|  |  |  |  |  |  |  |
| Married | 0.113 | 0.609 | 1006.4 | 128.6* | -420.2 | 457.7 |
|  | (0.0820) | (0.471) | (900.4) | (71.91) | (775.8) | (446.4) |
|  |  |  |  |  |  |  |
| Low socio-economic level | -0.0658 | -0.353 | 1735.2* | 76.79 | -2076.0** | -417.6 |
|  | (0.0600) | (0.439) | (908.7) | (52.65) | (838.3) | (318.4) |
|  |  |  |  |  |  |  |
| Cancer | 0.109 | 0.573 | -280.0 | 177.6*** | 842.2 | 384.6 |
|  | (0.0668) | (0.503) | (1029.2) | (43.61) | (959.3) | (346.8) |
|  |  |  |  |  |  |  |
| Worker | -0.00390 | -0.602 | 259.0 | 3.465 | -279.2 | -23.68 |
|  | (0.0589) | (0.393) | (670.9) | (54.58) | (657.6) | (306.1) |
|  |  |  |  |  |  |  |
| Constant | 0.273*** | 5.248*** | 1731.1 | -111.0* | -318.8 | 1523.3*** |
|  | (0.0962) | (0.631) | (1205.3) | (63.59) | (1163.9) | (501.2) |
|  |  |  |  |  |  |  |
| Observations | 59 | 59 | 59 | 59 | 59 | 59 |
| R-sq | 0.331 | 0.336 | 0.158 | 0.355 | 0.265 | 0.257 |

**Notes:** Estimates in each column correspond to the parameter of a linear regression with 57 degrees of freedom between the variables in the columns as dependent variable, and the variables in the rows as independent variables. Values for costs and net benefits are in USD dollars for 2018. For columns 5 and 5, we used the Colombian cost-effectiveness threshold (USD 5,180.8 per QALY) as the willingness-to-pay for one additional QALY. Robust standard errors are presented in parentheses.

**Appendix Table 2. Bootstrapped standard errors for the cost-utility analysis**

|  |  |  | Percentile  Confidence intervals | |
| --- | --- | --- | --- | --- |
| Parameter | Observed coefficient | Bootstrap std. Err. | LL | UL |
| INMB - All costs | 3204.921 | 1465.756 | 1028.802 | 6605.826 |
| INMB - Direct costs | 1134.733 | 461.4413 | 154.0285 | 2017.115 |
| ICUR - All costs* | -7784.146 | 45438.9 | -56043.08 | 1750.098 |
| ICUR - Direct costs | 589.9185 | 1087.153 | 174.7136 | 2381.788 |
| ICER MQoL - All costs* | -1242.912 | 2857.509 | -6732.861 | 327.3016 |
| ICER MQoL - Direct costs | 94.19359 | 104.9905 | 30.07743 | 310.6172 |

**Notes:** Standard errors were derived after 1,000 replications involving estimating differences in costs and health benefits (EQ-5D and MQoL). The INMB regressions involve a parameter of a single regression, while the ICUR and ICER were obtained as the ratio of coefficients coming from two regressions All regressions have 57 degrees of freedom and control for the presence of a catheter, gender, civil status, socio-economic level, diagnosis, and occupation of the patient.

* We present the output from the computation of the ICUR and ICER for transparency, yet, it is important to stress that these values are non-sensical given that the CI involve more than one quadrant of the cost-effectiveness plane (Hoch, J. S., Briggs, A. H., & Willan, A. R., 2002).^[[1]](#footnote-1)^ For this reason, our main interpretation comes from the INB.

**Appendix Figure 1. Robustness of Net Benefit Estimates**

Note: Estimates of the INMB considering alternative willingness-to-pay ($\lambda$) for computing net benefits under the total costs. The vertical line corresponds to USD 5.180, the Colombia cost-effectiveness threshold.

1. Hoch, J. S., Briggs, A. H., & Willan, A. R. (2002). Something old, something new, something borrowed, something blue: a framework for the marriage of health econometrics and cost‐effectiveness analysis. *Health economics*, *11*(5), 415-430. [↑](#footnote-ref-1)
